# Crystallization and structure of ebselen bound to Cys141 of human inositol monophosphatase

**DOI:** 10.1107/S2053230X20011310

**Published:** 2020-09-15

**Authors:** Gareth D. Fenn, Helen Waller-Evans, John R. Atack, Benjamin D. Bax

**Affiliations:** a Medicines Discovery Institute, School of Biosciences, Cardiff University, Cardiff CF10 3AT, United Kingdom

**Keywords:** inositol monophosphatase, IMPase, ebselen, bipolar disorder, tetramer

## Abstract

Inositol monophosphatase (IMPase) is inhibited by lithium, which is the most efficacious treatment for bipolar disorder. Several therapies have been approved, or are going through clinical trials, aimed at the replacement of lithium in the treatment of bipolar disorder. One candidate small molecule is ebselen, a selenium-containing antioxidant, which has been demonstrated to produce lithium-like effects both in a murine model and in clinical trials. Here, the crystallization and the first structure of human IMPase covalently complexed with ebselen, a 1.47 Å resolution crystal structure (PDB entry 6zk0), are presented. In the complex with human IMPase, ebselen in a ring-opened conformation is covalently attached to Cys141, a residue located away from the active site. IMPase is a dimeric enzyme and in the crystal structure two adjacent dimers share four ebselen molecules, creating a tetramer with approximate 222 symmetry. In the crystal structure presented in this publication, the active site in the tetramer is still accessible, suggesting that ebselen may function as an allosteric inhibitor or may block the binding of partner proteins.

## Abstract 2

A 1.47 Å resolution crystal structure of human inositol monophosphatase bound to the inhibitor ebselen is presented. In the structure, ebselen forms a selenosulfide bond to Cys141 and ebselen-mediated contacts between two dimers give a tetramer with approximate 222 symmetry.

## 1. Introduction   

Bipolar disorder is a chronic and debilitating psychiatric disorder that is characterized by cycles of mania followed by severe depression, frequently accompanied by bouts of psychosis. Although antipsychotic agents are the preferred short-term method of treatment, more efficacious mood-stabilizing drugs, such as lithium, are used in long-term clinical management (Geddes & Miklowitz, 2013 ▸). Lithium is the gold-standard treatment for bipolar disorder; however, it has several serious side effects, such as nausea and cognitive impairment, in addition to a narrow therapeutic window (Rybakowski, 2016 ▸). Because of these liabilities, other less efficacious mood stabilisers (for example lamotrigine) are now often used in the treatment of bipolar disorder (Won & Kim, 2017 ▸). One enzyme inhibited by lithium is inositol monophos­phatase (IMPase; Gill *et al.*, 2005 ▸), which has led to rational drug design targeting IMPase as a strategy for developing novel therapies for bipolar disorder (Brown & Tracy, 2013 ▸).

IMPase is a key enzyme in the intracellular phosphatidyl­inositol (PI) signalling pathway, whereby IMPase dephos­phorylates inositol 1-, 3- or 4-phosphate, collectively known as InsP1, to produce *myo*-inositol, also known as free inositol (Atack *et al.*, 1995 ▸). Cleavage of InsP1 into *myo*-inositol by IMPase is required for the recycling of inositol for subsequent use in the PI signalling pathway (Atack *et al.*, 1995 ▸). Inositol is an essential precursor for the synthesis of PI, which is subsequently utilized in the synthesis of phosphatidylinositol phosphates (PIPs). These include PI(4,5)P_2_, which is cleaved by phospholipase C following GPCR signalling to release the second messengers diacylglycerol (DAG) and inositol 1,4,5-trisphosphate (IP_3_) (Phiel & Klein, 2001 ▸). The observed depletion of free inositol and accumulation of the substrate of IMPase, InsP1, coupled with a reduction in agonist-invoked IP_3_ formation, in cells and animals treated with lithium led to the development of the inositol-depletion hypothesis to explain the mechanism by which lithium exerts its effects (Berridge *et al.*, 1989 ▸).

The inositol-depletion hypothesis suggests that lithium produces a reduction in free inositol primarily via blocking the recycling of inositol from InsP1, which leads to a decrease in PI(4,5)P_2_ and a slowing of the PIP signalling pathways that are postulated to be hyperactive in bipolar disorder (Harwood, 2005 ▸). Further evidence to support the inositol-depletion hypothesis comes from observations that the mood stabilisers carbamazepine and valproic acid also lead to the depletion of free inositol and attenuation of PI(4,5)P_2_ signalling pathways (Williams *et al.*, 2002 ▸). Therefore, targeting IMPase as a means of depleting free inositol is of scientific interest and led to the search for new IMPase inhibitors.

One such IMPase inhibitor is ebselen [2-phenyl-1,2-benz­isoselenazol-3(2H)-one], an organoselenium compound which functions as a glutathione peroxidase mimic (Nakamura *et al.*, 2002 ▸). Ebselen is believed to act through the reduction of reactive oxygen species (ROS) by binding covalently to cysteine residues or thiols to form selenosulfide bonds that lead to its pharmacological effect (Azad & Tomar, 2014 ▸). However, it is not known whether the covalent binding of ebselen to specific groups is directly or indirectly responsible for its mechanism of action (Ullrich *et al.*, 1996 ▸).

Ebselen has been demonstrated to inhibit IMPase in a covalent manner, with effects consistent with those of lithium, through depletion of free inositol in mouse brain (Singh *et al.*, 2013 ▸). Subsequent trials in a healthy cohort demonstrated that ebselen leads to decreased *myo*-inositol in the anterior cingulate cortex, in addition to effects consistent with the attenuation of PIP signalling (Singh *et al.*, 2015 ▸). At present ebselen is currently in a stage 2 clinical trial for the treatment of bipolar disorder; however, results from the trial have not been released at the time of writing.

Ebselen is known to bind to several proteins; crystal structures show ebselen covalently bound to cysteine residues of proteins including SOD1 (Capper *et al.*, 2018 ▸; Chantadul *et al.*, 2020 ▸) and the transpeptidase LdtMt2 from *Mycobacterium tuberculosis* (de Munnik *et al.*, 2019 ▸). Ebselen has also been reported to inhibit the main protease from SARS-CoV-2 (Jin *et al.*, 2020 ▸). These multiple targets suggest several potential therapeutic uses for ebselen, but also that there are likely to be off-target side effects.

Whilst inhibition of IMPase by ebselen has been demonstrated *in vitro*, confirmation of the ebselen binding loci and the exact mechanism of action on IMPase remain unclear. Structures of IMPase have been published with a variety of ligands, including a structure of human IMPase with the lithium mimetic L-690,330 (Kraft *et al.*, 2018 ▸). In this paper, we present a 1.47 Å resolution structure of ebselen covalently bound to cysteine residue 141 of human IMPase (PDB entry 6zk0). This is the first structure of IMPase to be published that demonstrates direct covalent binding of ebselen to IMPase.

## 2. Materials and methods   

All reagents were purchased from Sigma–Aldrich or Thermo Fisher unless stated otherwise.

### 2.1. Macromolecule production   

The IMPase construct was as described by Kraft *et al.* (2018 ▸) and *Escherichia coli* Rosetta2 (DE3) cells were used for IMPase production. A starter culture (10 ml) of transformed *E. coli* cells was grown overnight and was used to inoculate 1 l LB medium containing 2.5 m*M* betaine, 660 m*M* sorbitol, 35 mg ml^−1^ chloramphenicol and 50 mg ml^−1^ ampicillin. The culture was grown at 37°C to an OD_600_ of 0.8. IMPase expression was induced by the addition of 0.5 m*M* IPTG and the culture was grown overnight at 25°C.

The *E. coli* cells were pelleted by centrifugation at 9600*g* for 20 min at 4°C and were resuspended in 50 ml lysis buffer [20 m*M* Tris–HCl pH 7.8, 150 m*M* NaCl, 40 U ml^−1^ DNAse and one EDTA-free protease-inhibitor tablet (Roche)]. The resuspended pellet was lysed by sonication and the debris was pelleted by centrifugation at 33 000*g* for 20 min at 4°C. The clarified sonicate was heat-treated at 68°C for 1 h and the precipitant was pelleted by centrifugation at 32 000*g* for 20 min at 4°C. The supernatant was then incubated with Co–NTA resin at 4°C for 1 h. After incubation, the resin was washed with seven resin volumes (RV) of wash buffer (20 m*M* Tris–HCl pH 7.8, 150 m*M* NaCl, 15 m*M* imidazole) and the IMPase was eluted with 5 RV of elution buffer (20 m*M* Tris–HCl pH 7.8, 150 m*M* NaCl, 250 m*M* imidazole).

The eluted fractions were pooled and incubated overnight at 4°C with Pierce HRV 3C protease solution. This removes the N-terminal tag (MHHHHHHLEVLFQ) by cleaving at LEVLFQ↓GP (the sequence is given in Table 1 ▸). Cleaved IMPase was purified initially by incubation with Glutathione Sepharose 4 Fast Flow Resin that had been pre-equilibrated with two column volumes of size-exclusion chromatography (SEC) buffer (20 m*M* Tris–HCl pH 7.8, 150 m*M* NaCl). The flowthrough from the column was collected, concentrated to 5 ml and loaded onto a HiLoad 26/600 Superdex 75 Prep-Grade SEC column pre-equilibrated with SEC buffer. The proteins were gel-filtered at a flow rate of 1 ml min^−1^ and the fractions containing IMPase were pooled, buffer-exchanged into storage buffer [20 m*M* Tris–HCl pH 7.8, 150 m*M* NaCl, 1 m*M* EDTA, 10%(*v*/*v*) glycerol] and concentrated to 20 mg ml^−1^ using 10 kDa cutoff protein concentrators at 4000*g* prior to storage at −20°C. This protocol gave a typical yield of 2 mg IMPase per litre of culture.

### 2.2. Crystallization   

The crystallization of human IMPase was carried out using SWISSCI 3 Lens sitting-drop plates; the plates were set up using a Mosquito liquid-handling robot (TTP Labtech). To co-crystallize IMPase with ebselen, a 20 µl aliquot of IMPase at 20 mg ml^−1^ in storage buffer [20 m*M* Tris–HCl pH 7.8, 150 m*M* NaCl, 1 m*M* EDTA, 10%(*v*/*v*) glycerol] was incubated with 50 m*M* ebselen (6 µl of 200 m*M* ebselen in DMSO). The sample was transferred to a 500 µl microcentrifuge tube, placed on a roller and incubated at room temperature for 30 min prior to setting up crystallization plates.

A reservoir solution consisting of 0.2 *M* MnSO_4_, 0.1 *M* MES, 28% PEG 4000 pH 5.5 was added to the IMPase and ebselen solution in a 1:1 ratio and incubated at 20°C (see Table 2 ▸). Crystals appeared after seven days and continued to grow until they were harvestesd for data collection on day 14; they were cryoprotected by transfer into 20%(*v*/*v*) glycerol, 80%(*v*/*v*) reservoir solution and flash-cooled prior to data collection.

### 2.3. Data collection and processing   

An X-ray diffraction data set was collected from a single cryocooled crystal on beamline I04-1 at Diamond Light Source (Table 3 ▸). The data (2000 images of 0.1°) were processed with the *xia*2 pipeline at Diamond Light Source (Winter, 2010 ▸; Winter *et al.*, 2013 ▸) to give a 1.47 Å resolution data set in space group *P*3_1_21, with unit-cell parameters *a* = *b* = 84.02, *c* = 150.22 Å, α = β = 90, γ = 120°.

There are nine crystal structures of human IMPase in the PDB with similar unit-cell parameters, all of which belong to space group *P*3_2_21; thus, the data were re-indexed and remerged in *P*3_2_21. The data were merged using *AIMLESS* version 0.7.4 (Evans & Murshudov, 2013 ▸). The Rcp statistic, which is used to estimate cumulative radiation damage in *AIMLESS* (Diederichs, 2006 ▸), did not increase significantly over the 2000 frames.

### 2.4. Structure solution and refinement   

The structure was solved by rigid-body refinement using the 1.7 Å resolution crystal structure of IMPase with manganese (PDB entry 6gj0; Kraft *et al.*, 2018 ▸). Initial structure solution used the *DIMPLE* pipeline; this pipeline provides the user with a quick method to identify data sets that have a bound ligand or drug candidate in the crystal (http://ccp4.github.io/dimple/; Wojdyr *et al.*, 2013 ▸).

**Figure 1:**
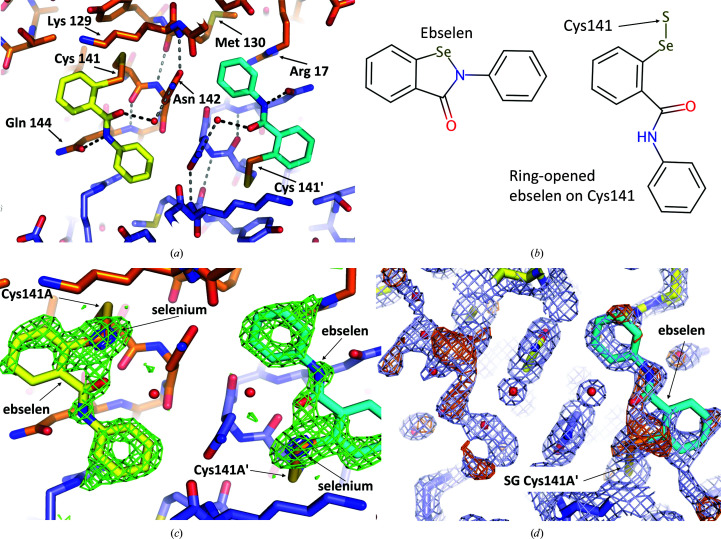
View of ebselen attached to Cys141 (PDB entry 6zk0). (*a*) Overview of two ebselen molecules attached to the *A* and *A*′ (symmetry-related) subunits around the crystallographic twofold axis. One subunit (*A*) has C atoms in orange and the second subunit (*A*′) has C atoms in slate blue (N atoms are blue, O atoms are red, Se atoms are orange and S atoms are yellow-orange). C atoms in one ebselen are yellow and those in the second ebselen are cyan. Hydrogen bonds near ebselen are indicated by dotted lines. (*b*) Chemical structures of ebselen and ring-opened ebselen on Cys141 (drawn with *Marvin*; https://www.chemaxon.com). (*c*) Final ebselen OMIT map (*F*_o_ − *F*_c_; 3σ, green; 15σ, blue). Note that the peaks on the seleniums (blue mesh) are 20.5σ and 19.6σ in this ebselen OMIT map. (*d*) Original *DIMPLE* (Wojdyr *et al.*, 2013 ▸) 2*F*_o_ − *F*_c_ map (1σ, light blue) and *F*_o_ − *F*_c_ difference map (3σ, orange). For subunit *A* the *DIMPLE*-refined structure with waters (small red spheres) refined into the density for the ebselen is shown. For the *A*′ subunit the ‘final’ coordinates (including ebselen) are shown.

Initial maps showed clear electron density (Fig. 1 ▸) for a single ebselen molecule attached to Cys141 in both subunits *A* and *B* of the dimer (the *P*3_2_21 cell has one dimer in the asymmetric unit). The ebselen was built onto Cys141*A* and Cys141*B* in *Coot* (Emsley *et al.*, 2010 ▸; Emsley, 2017 ▸). Restraints for the covalently bound ebselen were generated in *AceDRG* (Long *et al.*, 2017 ▸) and the structure was refined using *REFMAC*5 (Murshudov *et al.*, 2011 ▸).

Although the electron density was very clear for the terminal phenyl ring of the compound (Figs. 1 ▸ *c* and 1 ▸ *d*) and the electron-density maps have a large peak for the Se atom (covalently bonded to the S atom of Cys141), the electron density suggests that some radiation damage has occurred to the sulfur–selenium bond (Weik *et al.*, 2002 ▸; Garman, 2010 ▸). The data are consistent with a model in which some initial radiation damage occurred to the sulfur–selenium bond (within the first few degrees of data), after which a steady state occurred (the bond reforming after breaking owing to radiation; Gerstel *et al.*, 2015 ▸).

Each active site contains three Mn^2+^ ions in PDB entry 6gj0 (Kraft *et al.*, 2018 ▸), while in PDB entry 2bji (Gill *et al.*, 2005 ▸) each active site contains three Mg^2+^ ions. In our structure, site 2 (Gill *et al.*, 2005 ▸) does not have sufficient electron density for an Mn^2+^ ion (Mn^2+^ ions have 23 electrons). We modelled a similarly coordinated sodium ion at this site, because we had no Mg^2+^ ions in our crystallization experiment (the protein was provided in 150 m*M* NaCl and the crystallization buffer contained 200 m*M* MnSO_4_). However, we cannot rule out the possibility that this is an Mg^2+^ ion, rather than an Na^+^ ion (both Na^+^ and Mg^2+^ ions have ten electrons). This ‘site 2’ is the position in which lithium is postulated to bind with tetrahedral coordination geometry (Gill *et al.*, 2005 ▸). However, the co­ordination geometry in our structure is consistent with two Mn^2+^ ions and one Mg^2+^ ion, each with ‘standard’ octahedral coordination geometry. Most of the active-site metal atoms are modelled in two positions and have temperature factors similar to those of the surrounding residues (Masmaliyeva & Murshudov, 2019 ▸).

In the deposited structure (PDB entry 6zk0), the ebselens on Cys141 in subunits *A* and *B* each have an occupancy of 0.6, but the Se atoms are modelled in two positions (Supplementary Fig. S1). In the crystal, the selenium–sulfur bond has been modelled with an occupancy of 0.4 or 0.35 (see Supplementary Fig. S1). A second selenium position observed in the electron-density maps is some 1.3 Å further away from the S atom of Cys141 and this second position is likely to be caused by radiation-induced cleavage of the selenosulfide bond (Gerstel *et al.*, 2015 ▸). Refinement statistics are summarized in Table 4 ▸.

## 3. Results   

### 3.1. Human IMPase structure with ebselen bound at Cys141   

The 1.47 Å resolution crystal structure of human IMPase with ebselen (PDB entry 6zk0) was solved using a structure of human IMPase with the same unit cell and space group (PDB entry 6gj0; Kraft *et al.*, 2018 ▸). Electron-density maps (Fig. 1 ▸) clearly showed ebselen attached only to a single cysteine, Cys141. The binding of ebselen to the Cys141 residue in each monomer does not lead to noticeable changes in conformation in the active site that would prevent the catalytic activity of IMPase. Additionally, the binding of ebselen to Cys141 does not appear to prevent dimer formation, as shown by the dimers (and tetramers) present in this structure (PDB entry 6zk0). However, whilst this structure clearly shows ebselen bound to Cys141 in each monomer of IMPase, our structure does not rule out the possibility that Cys218 could be modified *in vivo*.

**Figure 2:**
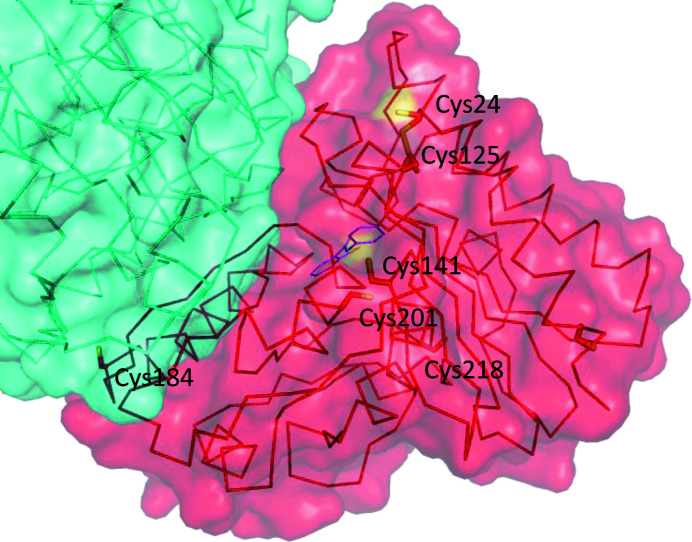
Cysteine residues in IMPase with ebselen bound to Cys141 (PDB entry 6zk0). IMPase is shown as a C^α^ ribbon trace and the side chains of the seven cysteines are shown as sticks on the ‘red’ subunit. The second subunit in the dimer is shown in cyan. A semi-transparent surface is shown; note that where the S atoms of the cysteine residues are on the surface of the protein, the surface is yellow (Cys24 and Cys141). Cys184 also has some surface accessibility in the monomer, but is largely buried at the dimer interface, so no yellow is visible for Cys184 in this figure.

IMPase contains seven cysteine residues, amino acids 8, 24, 125, 141, 184, 201 and 218; of these residues, only Cys218 is near the active site. Four of the cysteine residues are buried and would not be expected to be accessible to modification by ebselen (Cys8, Cys125, Cys201 and Cys218). Of the three cysteines that have some surface accessibility in the monomer, one of them, Cys184, is largely buried in the dimer interface, as shown in Fig. 2 ▸ (PDB entry 6zk0). The procedure used to co-crystallize ebselen with IMPase allowed partial oxidation to form a Cys141–ebselen selenosulfide bond (Fig. 1 ▸). In our structure Cys24 has some surface accessibility when reduced, but when oxidized forms a disulfide with Cys125 (Fig. 2 ▸). Cys125 has no surface accessibility whether oxidized or reduced. A partial Cys25–Cys125 disulfide is also observed in PDB entry 6gj0 (Kraft *et al.*, 2018 ▸).

Previous research suggested that Cys218 is the primary reactive cysteine residue (Knowles *et al.*, 1992 ▸), which is supported by the reduced inhibition of C218A mutant IMPase by ebselen (Singh *et al.*, 2013 ▸). In the structure reported here (PDB entry 6zk0) and other human IMPase structures Cys218 is largely buried and therefore seems to be an unlikely target for modification, as it is unclear how ebselen would gain access. If the side chain of Cys218 is modified by ebselen it would be likely to lead to a substantial reorganization of the protein structure; Asp220 coordinates an active-site metal ion.

From our structure, it appears that Cys141 is likely to be the primary binding site of ebselen. The sulfur group of this residue is exposed on the surface of IMPase (Fig. 2 ▸), and this residue is not in close proximity to another cysteine residue in either the monomeric or the dimeric form, and thus is unlikely to form a disulfide bond (PDB entry 6zk0). Cys141 is conserved in mammals (Knowles *et al.*, 1992 ▸; Singh *et al.*, 2013 ▸), and an analogous cysteine residue (Cys138) is present in *Staphylococcus aureus* IMPase. Given this level of conservation, it is probable that Cys141 is a functionally important residue in IMPase (Dutta *et al.*, 2014 ▸).

Cys141 has previously been shown to be a reactive cysteine residue, as demonstrated by its affinity for the thiol probes pyrenemaleimide (Greasley *et al.*, 1994 ▸) and *n*-ethylmaleimide (Knowles *et al.*, 1992 ▸). In this structure, ebselen is in an open-ring conformation, with the Se atom forming a selenosulfide bond to the sulfur group of Cys141, which is consistent with the known binding mechanism of ebselen (Capper *et al.*, 2018 ▸). Each monomer of IMPase in this structure has a single ebselen molecule bound to Cys141 (PDB entry 6zk0).

Whilst the conservation of Cys141 would suggest a critical role of this residue, its exact function remains unclear. The residue is not in close proximity to the active site, and therefore the residue is unlikely to be involved in catalytic activity. One possibility is that the residue may undergo post-translational modification *in vivo* and may be redox-active (Marino & Gladyshev, 2012 ▸), linking ebselen to therapeutic effects as a known antioxidant.

### 3.2. Ebselen-bound IMPase is observed to form a tetramer with 222 symmetry in the solid state   

**Figure 3:**
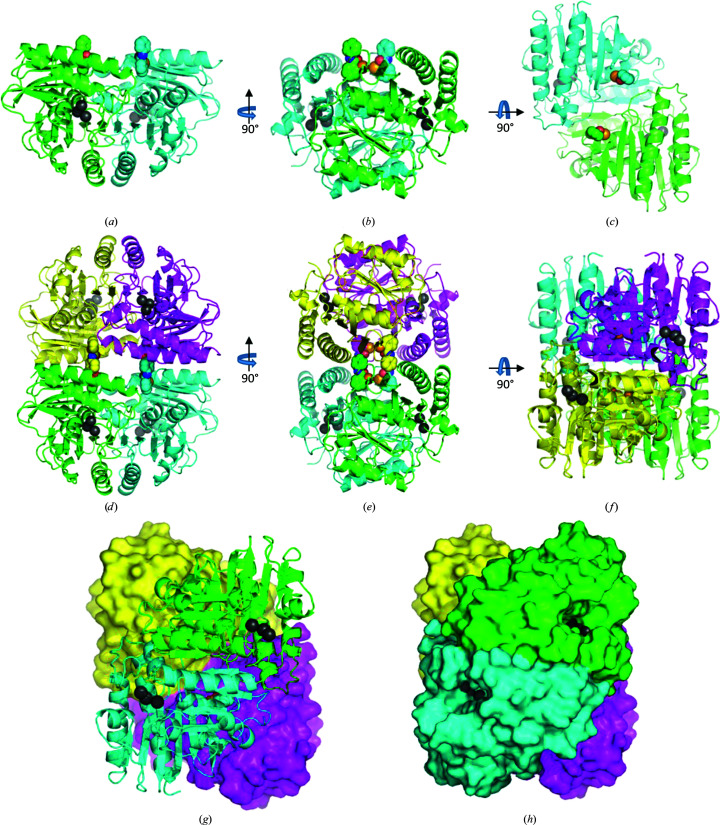
Orthogonal views of the IMPase dimer (and tetramer) showing ebselen on Cys141 and metal ions in the active sites based on the structure of PDB entry 6zk0. (*a*) The two subunits in the dimer are shown as green (subunit *A*) and cyan (subunit *B*) cartoons, with ebselen attached to Cys141*A* and Cys141*B* in space-filling representation. Metal ions (Mn^2+^/Na^+^) at each active site are shown as grey spheres. (*b*, *c*) Orthogonal views. (*d*, *e*, *f*) The same views as in (*a*, *b*, *c*) but also showing a second dimer related by a crystallographic twofold axis. Subunit *A*′ is in yellow and subunit *B*′ is in magenta. The view in (*e*) is along the crystallographic twofold that rotates the *AB* dimer (green/cyan) onto the *A*′*B*′ dimer (yellow/magenta). (*g*, *h*). Two views of the tetramer from underneath, showing that the three metal ions (grey/black spheres) at each active site are still accessible in the tetramer. In (*h*), a surface is shown for both dimers.

Fig. 3 ▸ shows the position of Cys141 on subunits *A* and *B* of an IMPase dimer. The two ebselen molecules covalently bind to each dimer and localize with two other ebselen molecules on a second dimer. The two ebselen molecules on each dimer increase the contacts with a neighbouring dimer, which gives a tetramer with approximate 222 symmetry in the crystal. However, as shown in Figs. 3 ▸(*g*) and 3 ▸(*h*), the active site still appears to be accessible.

Interestingly, other members of the IMPase superfamily, including fructose-1,6-bisphosphate (FBPase), have been observed to have both dimer and tetramer forms (Hines *et al.*, 2007 ▸). Tetrameric forms of IMPase have also been observed in the anaerobic hyperthermophilic eubacterium *Thermotoga maritima* (Stieglitz *et al.*, 2007 ▸).

## 4. Discussion   

The IMPase crystal structure that is presented (PDB entry 6zk0) has ebselen covalently attached to Cys141; however, the extent to which this binding brings about the inhibitory effect of ebselen on IMPase is not clear. It is possible that the modification on Cys141 is biologically relevant, and that this is a cysteine residue that is modified *in vivo* by ebselen, with binding over the previously suggested preferred residue Cys218 (Singh *et al.*, 2013 ▸). Cys141 has been shown to be a reactive cysteine residue (Greasley *et al.*, 1994 ▸), so it possible that ebselen binding at Cys141 causes inhibition of IMPase. However, the binding of ebselen does not affect the conformation of the active site or prevent dimer formation; with the high conservation of Cys141 across species, it is likely to be a functional residue with a potential regulatory redox role.

There is evidence that modulation of IMPase away from the active site and dimer interface can affect activity; synthetic peptides that disrupt IMPase–calbindin interactions prevent calbindin-mediated activation of IMPase (Noble *et al.*, 2018 ▸) and mediate antidepressant-like effects in mice (Levi *et al.*, 2013 ▸). It is possible that ebselen interferes with accessory protein binding, possibly by formation of the tetramer seen in the crystal, to moderate the activity of IMPase *in vivo*.

However, the possibility that the conditions used in crystallization do not reflect physiological conditions and that Cys141 and Cys218 could be modified differently by ebselen *in vivo* cannot be ruled out. The binding of ebselen to Cys141 does not appear to have significantly altered the structure of the dimer or the active site. Should the ebselen/IMPase tetramers observed prove to be biologically relevant, this would suggest a new mechanism for the regulation and subsequent inhibition of IMPase that could be utilized in the development of novel therapeutics.

## Supplementary Material

PDB reference: human IMPase bound to ebselen, 6zk0

Supplementary Figure 1. DOI: 10.1107/S2053230X20011310/uf5002sup1.pdf

## Figures and Tables

**Table 1:** Macromolecule-production information

Source organism	Human
DNA source	pET-15b vector containing human inositol monophosphatase with an N-terminal His_6_ tag followed by an HRV 3C cleavage site
Cloning vector	pET-15b
Expression vector	pET-15b
Expression host	*E. coli* Rosetta2 (DE3)
Complete amino-acid sequence of the construct produced	MHHHHHHLEVLFQGPMADPWQECMDYAVTLARQAGEVVCEAIKNEMNVMLKSSPVDLVTATDQKVEKMLISSIKEKYPSHSFIGEESVAAGEKSILTDNPTWIIDPIDGTTNFVHRFPFVAVSIGFAVNKKIEFGVVYSCVEGKMYTARKGKGAFCNGQKLQVSQQEDITKSLLVTELGSSRTPETVRMVLSNMEKLFCIPVHGIRSVGTAAVNMCLVATGGADAYYEMGIHCWDVAGAGIIVTEAGGVLMDVTGGPFDLMSRRVIAANNRILAERIAKEIQVIPLQRDDED

**Table 2:** Crystallization conditions

Method	Sitting-drop vapour diffusion
Plate type	SWISSCI 3 Lens crystallization plate
Temperature (K)	293
Protein concentration (mg ml^−1^)	20
Buffer composition of protein solution	20 m*M* Tris–HCl pH 7.8, 150 m*M* NaCl, 1 m*M* EDTA, 10%(*v*/*v*) glycerol
Composition of reservoir solution	0.2 *M* MnSO_4_, 0.1 *M* MES, 28% PEG 4000 pH 5.5
Volume and ratio of drop	100 nl, 1:1
Volume of reservoir (µl)	50

**Table 3:** Data collection and processing Values in parentheses are for the outer shell. Test data sets were also produced, merging the first 800 and last 800 images from the data set, to check for radiation-damage effects (see Section 2.4 for details). The analysis suggested that the best data set was obtained by using all data, and that although some radiation damage appeared to be present in the data, this damage was not reduced by removing the later frames from the data set.

Diffraction source	I04-1, Diamond Light Source
Wavelength (Å)	0.91188
Temperature (K)	100
Detector	PILATUS 6M-F
Crystal-to-detector distance (mm)	260.0
Rotation range per image (°)	0.1
Total rotation range (°)	200
Exposure time per image (s)	0.1
Space group	*P*3_2_21
*a*, *b*, *c* (Å)	84.02, 84.02, 150.22
α, β, γ (°)	90.0, 90.0, 120.0
Mosaicity (°)	0.080
Resolution range (Å)	45.0–1.47 (1.50–1.47)
Total No. of reflections	1155862 (53779)
No. of unique reflections	104886 (5118)
Completeness (%)	100.0 (100.0)
Multiplicity	11.0 (10.5)
〈*I*/σ(*I*)〉	20.4 (1.2)
CC_1/2_	1.0 (0.498)
*R*_meas_	0.056 (2.123)
*R*_p.i.m._	0.017 (0.652)
Overall *B* factor from Wilson plot (Å^2^)	22.2

**Table 4:** Structure solution and refinement Values in parentheses are for the outer shell.

Resolution range (Å)	24.44–1.47 (1.50–1.47)
Completeness (%)	100.0 (100.0)
σ Cutoff	N/A
No. of reflections, working set	99740
No. of reflections, test set	5081
Final *R*_cryst_	0.1734
Final *R*_free_	0.2011
Cruickshank DPI	0.0662
No. of non-H atoms	
Protein	3694
Ebselen†	49
Ligand	54
Water	416
R.m.s. deviations	
Bonds (Å)	0.014
Angles (°)	1.856
Average *B* factors (Å^2^)	
Overall	29.4
Protein	27.9
Ebselen	31.7
Ligand	45.7
Water	40.3
Ramachandran plot	
Favoured regions (%)	98
Additionally allowed (%)	1.5
Outliers‡ (%)	0.5

† Ebselen attached to Cys141*A* had only one atom with two positions (the Se atom). Ebselen attached to Cys141*B* had every atom in two positions. There are 16 non-H atoms in ebselen.

‡ Lys36 is the only residue (just) outside the allowed region in both subunits (φ = −100°, ψ = −110°).
