# Supplementary material for: Crystallization and structure of ebselen bound to Cys141 of human inositol monophosphatase
Source: Acta Crystallogr F Struct Biol Commun. 2020 Sep 15;76(Pt 10):469–76. doi: 10.1107/S2053230X20011310 (PMC7531247; doi:10.1107/S2053230X20011310)
Supplement: Supplementary file 1 [file f-76-00469-sup1.pdf]

STRUCTURAL BIOLOGY COMMUNICATIONS

Volume 76 (2020)

Supporting information for article:

Crystallization and structure of ebselen bound to Cys141 of human inositol monophosphatase

Gareth D. Fenn, Helen Waller-Evans, John R. Atack and Benjamin D. Bax

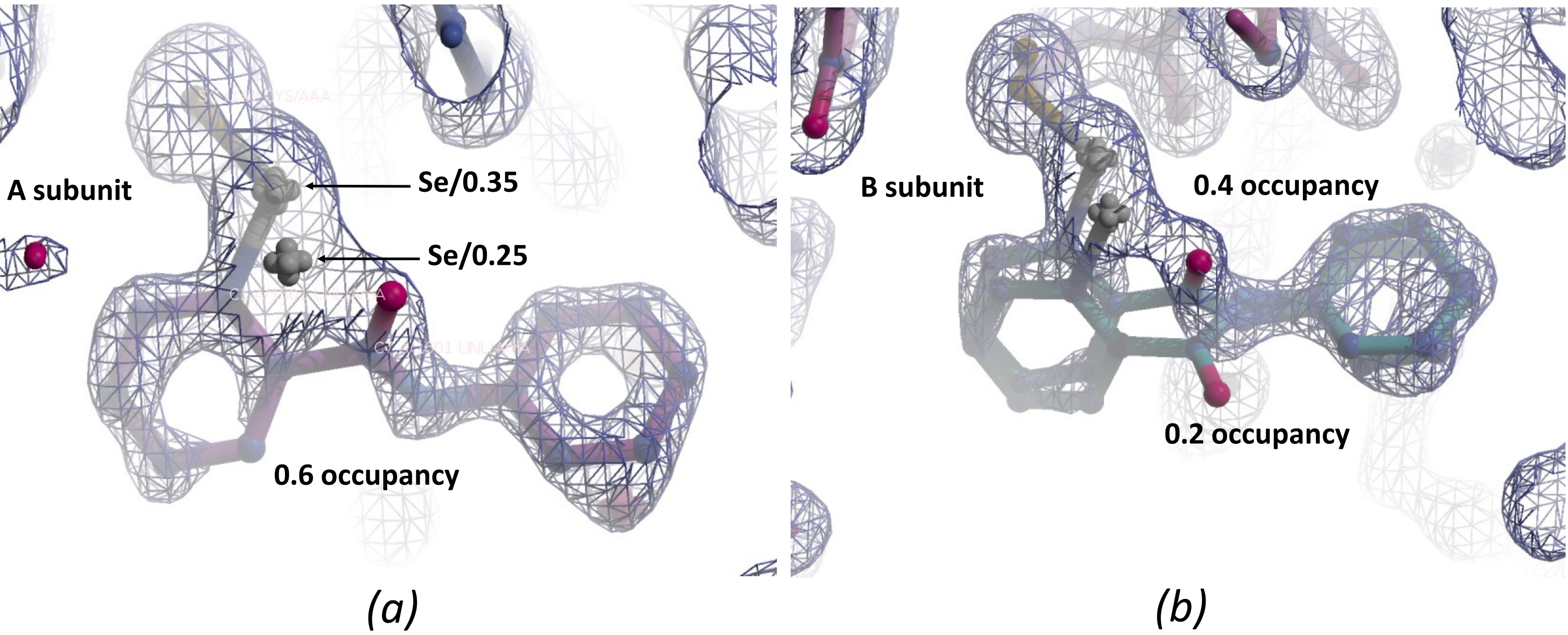

Supplementary Figure S1. Final models of ebselen attached to Cys141 in subunits A and B (PDB entry 6ZKO). *(a)* Final model for subunit A has the ebselen modelled in a single position with occupancy 0.6 for all atoms except the selenium. The selenium covalently attached to the sulfur of Cys141A has an occupancy of 0.35, but a second position of the selenium with occupancy of 0.25 is also modelled. The final 2Fo-Fc map is shown contoured at 1.1 sigma (0.33 electrons/$Å^3$). *(b)* Final model for subunit B has the ebselen modelled in two positions. That with occupancy 0.4 has the ebselen covalently attached to Cys141B. The second modelled conformation, with occupancy 0.2, has the selenium some 1.3 Å further away from the sulfur, presumably due to the radiation damage.
